# Upper Secondary School Pupils’ Experience of a Lifestyle Plan Based on Physical Power, Mental Harmony, and Social Capacity

**DOI:** 10.3390/ijerph20054532

**Published:** 2023-03-03

**Authors:** Fredrik Lygnegård, Marie Alricsson, Anna Hafsteinsson Östenberg

**Affiliations:** 1 Department of Natural Science and Biomedicine, Jönköping University, 551 11 Jönköping, Sweden; 2 Department of Sport Science, Linnaeus University, 352 95 Kalmar/Växjö, Sweden

**Keywords:** adolescents, health promotion, motivation, physical activity, physical education, web-based, youth

## Abstract

Purpose: This study aimed to illustrate upper secondary school pupils’ experience using a self-administered web-based health-promoting tool, the Swedish Physical Power, Mental Harmony, and Social Capacity (FMS) student profile. Method: Five upper secondary schools in Sweden were included. Focus group interviews with pupils (10 girls, 5 boys, 15–19 years) were conducted, and data were analyzed using qualitative content analysis. Result: Two themes were generated from six categories: a sense of participation and self-control of health: everyday well-being, objective formulation, disappointment, health awareness, limitations, and health-promoting change. The participants experienced that using the FMS made them aware of factors that influence their health. They also reported that being given feedback visually from the FMS, peers, and staff involved in the school was beneficial in increasing their motivation to maintain a health-promoting change regarding physical activity and lifestyle factors. Conclusion: The use of a self-administered web-based health-promoting tool is viewed as beneficial for raising awareness and motivation to implement strategies that help attain a healthier lifestyle in upper secondary school students regarding factors affecting perceived health.

## 1. Introduction

Mental illness has recently been seen to increase, with a higher prevalence among girls than boys in primary school. This has been stated as a factor that may affect school performance negatively [1,2]. There are several factors that can affect the subjective experience of health or illness, including socio-economic factors, accessibility to activities, and the extent of physical activity.

Self-determination is considered fundamental for promotion of motivation. This, in turn, is something that can contribute to increasing student influence regarding education. Deci et al. refer to the theory of self-determination (SDT), where three basic needs: competence, relatedness, and autonomy (self-determination), are fundamental in SDT [3]. The importance of motivation when it comes to physical activity is an established area of research in physical education, where one of the theoretical frameworks is SDT. According to SDT, an individual’s motivation is more likely to be diminished if the sense of competence, relatedness, and self-determination is reduced [4]. The theory has become more and more popular in research related to physical education, where the relevance is to connect theoretical tenets of SDT with practical physical education. Two variants of motivation are described in this framework: the internal (intrinsic) and the external (extrinsic). Intrinsic motivation is considered as the most autonomous form [4,5]. From this reasoning, it could be beneficial to promote the presence of autonomic motivation with the aim of achieving long-term behavior that increases health benefits for increased quality of life.

There is well-stated evidence that there are health benefits related to a physically active lifestyle. Some of these benefits have been shown to progress from adolescence to adulthood. To obtain these positive outcomes from regular physical activity, international guidelines have been recommended. Adolescents are recommended to implement daily physical activity lasting 60 min, at an intensity level corresponding from moderate to vigorous [6]. It has been found that the extent of physical activity in young people tends to be reduced during adolescence [7], such as decreased participation in associations, sports, or general physical activity in everyday life. The degree of physical activity, participation in exercise, or sporting events has been shown to be positively associated with level of self-esteem [8]. This reduction in everyday physical activity has been noted globally but is more evident in high-income countries, for example, in Europe and North America. There is also a difference between sexes, where data indicate a greater physical inactivity among girls [9]. A consequence of this might be related to a negative impact on life satisfaction and general well-being [10].

Physical activity is one of several factors that are important for good health in teenagers and affects body weight and sleeping habits [11]. These are factors that may affect an individual’s perceived self-esteem, self-confidence, and contribute to a sense of coherence and participation in different social contexts [12]. 

The increased sedentary behavior is a global phenomenon, and the number of adolescents who do not achieve the recommended frequency of physical activity for school-age adolescents has gradually increased in recent decades [9,13]. The reduced level of physical activity is something that can be assumed to be an influencing factor when it comes to academic performance. Evidence shows that implementation of physical activity in the education curriculum promotes academic performance in school [12,14,15,16,17,18], and more significantly for boys than girls [1,18]. Singh et al. show that the positive effect of increased physical activity in education is most strongly related to mathematics. However, when assessing language skills and the impact of physical activity, the level of evidence is not as convincing [19]. From this, an argument can be made that there are potential health benefits in encouraging and facilitating the performance of physical activity to promote a healthier lifestyle. 

Positive changes from a health perspective regarding lifestyle factors increase the chances of goal achievement if the goals are meaningful, reasonable, and measurable [20]. To increase understanding to contribute to health-promoting changes regarding individuals’ behavior related to physical activity, different models are used in health-related areas [21]. A commonly used model is the Transtheoretical Model (TTM), developed by the researchers Prochaska, DiClemente, and Norcross, who have analyzed and compared different theories in psychotherapy. The TTM comprises different steps that consider where an individual is positioned regarding a behavior change process. It starts from a stage of tendency to change to the fact that the individual maintains the change and normalizes it [21]. To achieve goal fulfillment, it is beneficial that a sense of how to reach the goal is present and that the individual has the opportunity to carry out the activity to reach the goal. This can be defined as executive control [22]. In a study by Nielsen et al., they concluded that physical activity has an impact on schoolchildren’s social relationships, motivation, well-being, and learning, stating that the pedagogical model is of importance [23]. To promote individuals’ self-management regarding health, different variants of digital tools have been developed in recent decades, such as the use of mobile and web-based applications [24,25].

The self-administering and web-based health tool Physical Power, Mental Status, and Social Capacity (FMS, Fysisk kapacitet, mental hälsa och social förmåga (in Swedish), now Bwell) has been used in upper secondary schools in Sweden for a couple of decades to help students to take their own responsibility and initiate any health-improving changes [26,27]. A more detailed description of FMS can be found in previously conducted studies [27]. Based on this background, an argument can be made that there are potential health benefits in encouraging and facilitating the performance of physical activity to promote a healthier lifestyle. This study therefore aimed to illustrate upper secondary school pupils’ experience using a self-administered web-based health-promoting tool, the FMS student profile. 

## 2. Materials and Methods

This study is part of a project where the aim has been to map and highlight upper secondary school pupils’ lifestyle. The participants have recorded data with a tool developed by the Swedish Institute of Physical, Mental, and Social capacity for adolescents between the ages of 12 and 19 years old. The tool is constituted by a questionnaire related to physical activity and behavioral factors, fitness tests (aerobic, anaerobic capacity, agility), and anthropometric proportions. Relevant school personnel and participants were informed regarding the interviews. 

Focus group interviews were conducted as a qualitative approach with the content analysis framework. This is a suitable method, according to Kamberelis et al., to investigate whether there are common denominators, patterns of opinion, or experiences among the participants [28].

The participants were pupils at upper secondary level recruited from five schools in southern Sweden. A subjective selection procedure was used. It was considered appropriate since the participants were relevant respondents regarding the research questions [29]. During their schooling, all participants had used the digital health tool FMS (now Bwell) student profile, sometime between 2018 and 2021. This formed the basis for the study’s research questions. A total of 21 (15 females, 6 males (age 16–18)) pupils were contacted, with 6 pupils not attending. In total, 15 pupils, 10 females and 5 males, were interviewed in 5 focus groups, respectively. The number of participants in each group varied between 2 and 4 participants. Interviews were carried out digitally because, during the period of data collection, there were national restrictions due to pandemic conditions related to high societal contagion of COVID-19 limiting interaction in real life [30].

**Figure 1:**
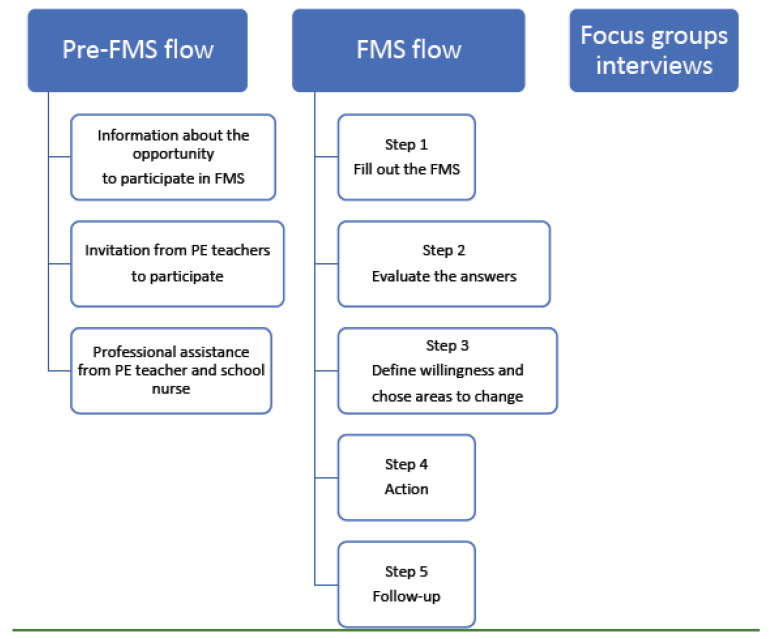
Flow chart showing the flow of FMS Swedish Physical Power Mental Harmony and Social Capacity (FMS) Student profile.

The health tool is an instrument designed to assess the physical, mental, and social health of adolescents in the age range of 12–19 years old and is a web-based, reliable, and self-administering instrument [27,31]. The purpose of the tool is to stimulate the pupil’s subjective involvement and awareness related to lifestyle factors, health, and well-being. The instrument is meant to facilitate pupils’ willingness to improve their physical, mental, and social health [31]. The definition FMS is a derivation from the World Health Organization’s (WHO) definition of health: “Health is a state of perfect physical, mental and social well-being not just the absence of disease” [32]. Users independently administer and record data regarding specific physical tests as well as anthropometric data [27]. In FMS, the user can also make a self-assessment of lifestyle factors and lifestyle habits. Based on the self-assessment and tests, the pupil has a conversation with teachers in sports and health or school nurses regarding the outcome. Conversations are also held about the pupils’ chosen goals when it comes to change/improvement/maintenance of lifestyle factors and lifestyle habits. The user is encouraged to take responsibility for variables that pose a risk for ill health, according to the FMS, which can be improved. FMS is a holistic concept that is anchored with the principal at each school and is used in collaboration with student health and teachers related to sports and health (Figure 1).

Recruitment of the participants was carried out with the help of a contact person in the respective schools. Participants were then contacted and informed via e-mail regarding the formality related to the communication platform where the digital meeting took place. The digital meeting was conducted using the video telephony software Zoom (zoom.us accessed on 25 January 2023). Focus group interviews were performed during autumn 2020 and spring 2021. All interviews were conducted by the same interviewer and lasted between 30 and 45 min, and all interviews were audio recorded. The interview guide was created with inspiration from subject areas that the participants encountered when creating and implementing the lifestyle plan in the web-based health tool.

Information regarding procedures and the aim of the study was given in writing and verbally to the participants. Consent was given by the participants to participate in the study. The present investigation was conducted in accordance with the Declaration of Helsinki for human studies and was approved for retrospective and prospective data by the Swedish Ethical Review Authority. The study is part of a project with approval from the Ethical Committee (Dnr 2018/244-31).

A qualitative content analysis was conducted, which is considered a useful method in a varied spectrum of areas that creates space for abstraction and interpretation at different levels [28]. The method is considered appropriate as it aims to highlight experiences and visualizes any similarities or differences when using a web-based health instrument. It can be assumed that there are different individual interpretations of reality [33]. The analysis process was performed by the creation of meaning units. These are the meaning-bearing parts of the text, which, in the next step, were condensed into shorter and more manageable text. The condensed text was given an abstracted but textual code, which was sorted into sub-categories and categories (Table 1). Throughout the analysis process, the codes were checked and compared for placement under the appropriate subcategory and category.

## 3. Results

The results demonstrated that the participants’ experience of the web-based health tool FMS can be grouped into two themes: sense of participation and autonomy regarding health. These two themes are defined by six categories (Table 2). The theme is a product of the re-contextualization of codes–sub-categories–categories. Where the codes were abstracted from every participant’s answer, perspective and discussion that often differed but could be abstracted into similar codes was eventually interpreted into the overall theme “sense of participation and autonomy regarding health”.

The result shows that the participants see benefits but also limitations when using the FMS tool. This applies, for example, to the experience that improvement habits and lifestyle factors were highlighted and clarified. Consequently, it led to an increased awareness of the relationship between perceived health and school performance. 

### 3.1. Sense of Participation and Self-Monitoring of Health

#### Satisfaction

The recipients stated that the use of the health tool increased an awareness of their individual lifestyle factors and lifestyle habits and that it gave an “aha” feeling and made it clear to them that there are areas of improvement that, according to the health tool, can affect their experience of health and performance in school.

E5: “I think I started thinking about being more physically active with that…but I think it opened quite a bit, you got a little bit like, yes it’s a little fun, when you feel like you’re evolving.”

A3: “I’ve noticed a difference since I started with this. Now, I’m not as tired and I can concentrate better. I had also set a goal of not snacking, but I still do. It’s so hard when I’m not deciding what to eat. It would have been easier if I had lived by myself, but then my second goal around sleep would be at risk and then that goal felt more important.”

The results show an insight among the participants that lifestyle factors, social factors, and physical activity are of importance when it comes to the subjective experience of health and ill health:

L1: “Health, I relate a lot to the fact that you have a good variety in the training and that you perform training in different ways. But also that you have a good diet and good sleep routines, that it is a good combination of everything. So that it’s not just about training. But it’s also about diet and sleep and such. So, you feel as good as possible.”

The results also show that there was an understanding and opinions about what might affect health. An increased awareness and sense of the opportunity to influence the outcome increased the sense of meaningfulness in the implementation of health-promoting tasks:

R4: “I think it depends on what your everyday life looks like. So if you sleep well, if you have something to look forward to, leisure interests, then it automatically improves your health, and if you do not have those parts of your everyday life, there will be more ill health.”

L5: “[…] in the past, there has not been so much strength training; it’s been more like, no training whatsoever; it’s been more for sports […] if so, no workout at gyms or similar, but after this, I learned how agile I was and what strength I had, and little else like that, which made me start to take more interest in it afterwards.”

In addition, it was experienced that the work with lifestyle plans increased curiosity, which, in turn, increased interest in implementing the changes that the participants chose to focus on. Increased interest regarding physical literacy or the importance of regular sleeping routines can be assumed to have increased motivation, which can affect the result in a positive direction for the participants.

### 3.2. Goal Formulation

With the use of the digital health tool, lifestyle factors were clarified for the participants, both visually and verbally. It was helpful to formulate a goal to work towards. In the focus groups, this enlightenment was somewhat surprising, but not entirely unexpected. The results also showed that the identified areas of improvement were already known to the participants:

O4: “I worked more on my mental health, so it was mostly that I tried to talk to others and say what I feel and so on. I didn’t do that before; I tried to work on it all the time.”

A3: “One of the plans was that I would sleep better, and I noticed that by the fact that I was tired all the time. Then I decided to plan for this, because I felt like I was tired, and it led to me not being able to do anything and it wasn’t good. I have written up what I will do and when I will do this task. My plan was first supposed to be carried out in the summer holidays, because I don’t like to get up so early, but then I decided to try doing it earlier and it went well.”

R4: “I’ve also been working on my fitness and it’s improved, but it’s not like I’ve done it just because I set it as a goal. […] I have felt that I need to work on that […]; it’s not like I’ve been thinking actively about what I set as a goal, a little more like a must, kind of.”

### 3.3. Disappointment

The results showed that goal achievement, according to the lifestyle plan, was inhibited by various factors. It could be about lifestyle factors, such as sleep and dietary habits, or that participants during the time when the work according to the lifestyle plan was going on suffered from illness or injuries. It also emerged that previous injuries related to the musculoskeletal system could be an obstacle. An unforeseen factor that arose during the implementation of the project was a global pandemic related to the spread of the virus COVID-19, which meant restrictions in everyday living:

E2: “[…] when Corona came, all sports were shut down and then, it became more difficult to train; you didn’t get the training you needed. Instead, you lay there at home and relaxed, which was not entirely good, because you must make up for that later. So, you have to blame yourself a little bit too that you didn’t take that run like you should have been doing, but so it’s mostly Corona, I would say. It limits!”

M4: “I would say it’s the thing with screen time, that it’s been hard to achieve the goal now that we’ve switched to distance education.”

C1: “[…] when I did too many knee exercises, my knee protested and then it was really like I had to stop. Because when my joints are protesting, I can’t do much more about them. Then I almost have to rest a week before I can start with anything else. I had to check how hard I pushed my body, so I didn’t go too far.”

L1: “I remember […] that I had a sore throat. I often get it in the fall […] So some running sessions I had to ignore; if it hadn’t been a sore throat, I might have succeeded better with my goals. Because I could have completed my workouts, properly so to speak. Uh so, it still went decently, but there was something that stopped me in a way.”

### 3.4. Health Awareness

The results showed an increased insight into lifestyle factors, and the importance of lifestyle habits related to larger contexts such as schoolwork. This can be related to, e.g., conversations between classmates, and feedback from involved teachers, school nurses, or mentors about lifestyle plans:

N3: “[…] I know that in our class, at least among those who were close friends and so on, you were more open, especially mental illness. But, in general, how are we really feeling; at least in my class, we were open with it; there’s nothing wrong with that, and in order for you to get better, you need to talk about it.”

E3: “The PE teacher follows up on class time, and then the mentors will become more involved and be able to elaborate on mentor talks. It also becomes possible to work on certain parts within the class, on things that need to be improved.”

The visualization of improvement areas identified with the help of the health tool increased the motivation for the participants in combination with feedback. This took place in the education context and during conversations with classmates. Feedback could also be received from other staff members at the school, for example, the school nurse/mentor/counselor.

E2: “[…] I already knew that my fitness was bad, […] you get it in black and white, that you do something about it.”

H1: “It was confirmed that this was good for the sake of one’s studies, the concentration, and I don’t know, I haven’t done a private survey on my grades or my results if they were improved thanks to this lifestyle plan back then, but I can still say that I had better focus during the lessons. […] it is a known fact that you get more energy from exercising […] and I actually felt that in class, you got more energy.”

### 3.5. Limitations

When the participants carried out the work related to their lifestyle plans according to the health tool, there was an ongoing global pandemic. The results showed that this affected the implementation of lifestyle plans because access to training facilities and social interaction opportunities were limited due to the restrictions. The participants believed this affected their motivation, for example. Motivation proved to be an important factor in experiencing meaningfulness in the implementation of improvement measures included in the participants’ lifestyle plans and that it may affect the outcome from a long-term perspective:

H2: “Yes, but also that. That is, always before, you have always had the practices has always been the basis for being at your best at the matches, but now that you can’t play games, it’s kind of, damn, boring to train. Thus, my motivation disappears because when I’ve been to school and come home, then you are quite tired, before it has always been that you have to train because otherwise you will not get to play the matches. Now you come home instead, and then you lie in bed for an hour or fall asleep a little or that you sit down and play or watch a football game on tv. I have allergies as well, so I get pretty tired quickly once I relax and I get tired. So that yes, no, is probably a bit of slothfulness.”

C1: “I noticed on my, I have pretty good sleep routines, but not right now. When we went over to distance education, it got worse. You had to sit in front of the computer a lot more, […] I had a harder time sleeping.”

When analyzing the interviews, an assumption can be made that after the implementation of the improvement measures and their evaluation, there was an increased understanding of the importance of continuity of work with factors such as physical activity, sleep, and diet that affects the sense of perceived health:

E5: “It has fallen between the cracks unfortunately […]. When we were in it, it felt like a task, which was for a certain period, it didn’t feel like now we’re doing it […] for the rest of our lives. I think we all gave up a little bit […] it didn’t come to, now we continue!”

The results showed that there was an insight regarding physical activity and the influence of lifestyle factors on health from the participants, but also that it is difficult to maintain continuity, for example, regarding exercise. The importance of the environment and the possibility of social interaction were highlighted in the focus groups. Participants discussed the importance of working with the lifestyle plans as part of the school being facilitating and motivating, which became more difficult when access to school facilities was limited by restrictions imposed at the national level due to the spread of COVID-19. 

### 3.6. Health-Promoting Change

Using the lifestyle plan made it easier to work on achieving the goals established in the lifestyle plan. The results showed that the participants experienced that physical activity, diet, and sleep contributed to increased well-being when they were more active, thinking about diet and sleep routines. When the goal was set and became visible in the lifestyle plan, it became easier to work toward goal achievement:

E5: “I think it was good that I set a goal, because if you had only trained to train, you wouldn’t have been able to, like, well, why am I doing this. But when setting a goal, then it became like, no, but now let’s do this. Now we have set a goal anyway, so my experience I thought was good; so you still got some routines for things.”

N3: “I’d say, eh, it’s been both easier in a way and harder, due to the fact that I had the goal of improving my mental part, that’s pretty obvious. That it affected the sedentary behavior and so because it is, it’s you haven’t been able to mentally, but once I start moving more, I’ve become more active mentally as well. They’ve kind of helped each other. I want to move, but it’s the mental health that’s involved…”

The results showed that despite the fact that there was an awareness of physical, mental, and social health that forms the basis of the lifestyle plan. The use of lifestyle plans facilitated the work to achieve goal fulfillment:

T2: “Yes, I think I did it a little bit, you know, those that you noticed were a little worse maybe, well, got a thought that you should work it up a bit… get it to the level with those that were good.”

## 4. Discussion

The purpose of this study was to investigate upper secondary school pupils’ experience regarding the use of a web-based health tool to promote increased well-being. By using the web-based health tool (FMS, now Bwell), pupils constructed individual goals that formed part of a lifestyle plan based on physical tests and survey questions. A qualitative content analysis of conversations in focus groups generated two themes: a sense of participation and autonomous control of health. The categories that form the basis of the two themes show that there is an understanding and knowledge, to some extent, that there are several variables which affect an individual’s perceived health, but also that it is challenging to maintain activities and lifestyle factors in keeping with what is positive for good health. Similar results were noted in the study by Sagatun et al., where students and involved staff felt that conversations regarding health factors were facilitated using a web-based health questionnaire [25].

Adolescence is a transition from childhood to adulthood, which means that more responsibility from society is placed on the young individual and less on their parents for any changes to occur in life. It becomes important for the teenager to become more and more “independent” in terms of well-being. Independence can be related to acquiring social skills and behavioral patterns that can be fundamental for obtaining health and well-being [34]. 

Focus group discussions, as a research method, have been used as a complement to quantitative data analysis, e.g., from surveys in several research areas. Examples include healthcare, pedagogy, and the market economy [35]. The focus group method is considered appropriate as the current study aims to investigate possible variations of the participants’ experiences when using the web-based health tool (FMS) as well as their experiences regarding any changes that may be related to this work. When conducting the focus group discussions, the same moderator was involved. This is considered to strengthen the reliability of implementation as it makes it easier for the participants to relate to the current division of roles in the discussion. Execution and documentation of focus groups have been undertaken via a digital platform, which meant that the indirect assessment of communication has been affected; for example, nonverbal communication such as body language, mimicry, etc., which is noted and occurs differently in conversations conducted in a common physical environment. During the period that interviews were conducted, a global pandemic was ongoing, which meant that there were nationwide restrictions that affected how the interviews were conducted. 

In the focus groups, it emerged that the participants found the feedback from the tests and questions in the health tool relevant and that it gave the participants a sense of increased awareness regarding their health. This is consistent with results from previous studies. Sagatun et al. examined the usefulness of web-based health information to support consultation between school healthcare and primary school pupils in Norway [25]. In their study, it emerged that when adolescents are given feedback by answering health-related questions, the experience is that it increases the pupil’s awareness of their own behavior. This is related to autonomous control to promote good health and well-being. A complete understanding of the relationship between lifestyle factors and perceived health is not self-evident. In an interview study conducted in Norway, a focus group interview was conducted with nurses linked to school health regarding their experience of pupils’ participation in physical education in secondary school. What the school nurses reported were, among other things, that they felt some students did not understand the connection between eating habits, bodily functions, and physical fitness [2].

When analyzing the discussions and opinions from the focus groups, it is noted that when it comes to issues concerning the private and personal sphere, students prefer to keep to her or himself or with classmates who are considered very close friends. Similar results were found in the study by Sagatun et al., namely, that some issues were perceived as uncomfortable, and more challenging to talk about openly [25]. 

The results also showed that the participants did not fully see the work carried out within FMS as something that could be continued in the long term. The findings further indicate that a normalization of the health-promoting changes according to the lifestyle plan has not taken place fully as the work based on the lifestyle plan has been completed. Based on the TTM, change is seen as a process that develops over time and is defined as six different stages of change: precontemplation, contemplation, preparation, action, maintenance, and termination stage, where termination means that the individual has fully adapted and practices change without the risk of falling back to the original behavior [21]. The importance of motivation emerged from conversations in the focus groups. From the results, it can be interpreted that motivation is important in a behavioral change toward increased well-being. Specific examples of motivational factors were given, such as being prepared for competition, feeling more mobile, or having more structured diet/sleep routines. These are examples of factors that are of importance for maintaining motivation [4,5]. During the period in which the participants carried out their work in accordance with the FMS, national restrictions were in place because of a global pandemic [30]. It became clear that the participants experienced this as a limiting factor that had a negative impact on motivation as social contacts were reduced and access to training facilities was restricted. The additional impact that emerged in the interviews was that this also contributed to changes in behavior, which, to some extent, made work more difficult according to the lifestyle plan. Examples of this could be sleep routines, eating habits, and screen time. This influence of motivation can be based on the SDT, formulated by Deci et al., relating to what the underlying factors (internal or external) are regarding the change of motivation [4]. For example, training to get a place in the team before a match constitutes a form of external motivation. The authors argue that external motivation is dependent on other factors, such as team affiliation. Internal motivation, on the other hand, is related to the individual reaching an insight into meaningfulness on his or her own, where it is he or she who keeps the motivation up to complete the activity. From the results, it appears that when social activities were restricted because of the COVID-19 restrictions, it resulted in more inactive behavior, as well as an increase in screen time. 

A prerequisite for a goal to feel meaningful, and that it increases a sense of meaningfulness and participation, is that the goal is possible to achieve and that it is reasonable [20,36]. The fact that the participants’ work and results were clarified via FMS and through conversations with teachers was highlighted in the conversations as positive. The participants received confirmation that they became more mobile, improved fitness, started talking to others about feelings, or created healthier sleeping habits. This result can be related to the stages of the transtheoretical model regarding a change in behavior linked to perceived health. It is only when the individual becomes aware of the benefits regarding change of behavior and can weigh them against what will be required of the individual that a change of behavior has an increased chance of being long-lasting [21]. This is what has been demonstrated as contributing factors in facilitating an increase in the individual’s well-being in previous studies [25]. The impact of the ongoing pandemic on participants’ experiences regarding creation and execution of lifestyle plans cannot be ignored. The result shows that there were challenges, e.g., when training in social contexts was limited to reduce the risk of spreading COVID-19 at the societal level. For the same reason, access to premises where physical activity is practiced was restricted. Included in the national strategy to reduce community contagion was, among other things, to limit teaching that was carried out at the school and instead switching to distance education. Participants who had goals related to sleep, screen time, and diets pointed out that it became more demanding to get into the routines that were a prerequisite for goal fulfillment according to the lifestyle plan.

Limitations of the study are related to the number of participants in respective focus groups. This can be an influencing factor when it comes to revealing shared experiences that are multifaceted when it comes to the discussed subjects. If there are fewer participants, this dynamic is of course not always possible. In the current study, there was only one of a total of five focus groups having two participants. This focus group, however, was surprisingly the one with the highest level of dynamic interaction between the participants, offering rich data material regarding participant experiences. That it was the same moderator in each focus group can be seen as a limitation, as it can be beneficial that analysis of collected data includes additional assessors, which can contribute to a more comprehensive overall picture through discussion and reflection [33].

## 5. Conclusions

The results of the study show that the participants’ experience of using a self-administered health tool has a promoting impact on goal achievement, in terms of independent engagement regarding perceived health. Encouraging pupils and giving them tools to visualize and concretize health-promoting changes in everyday life can be seen as beneficial for strengthening pupils’ autonomous impact of their self-perceived well-being and health. The results of this study indicate that by increasing awareness of lifestyle factors, in relationship to well-being, was experienced as positive by the participants. This can be a facilitating factor to obtain a more long-term health-promoting change due to strengthening of the pupils’ participation and involvement in their effort to reach their self-determined goals.

## Figures and Tables

**Table 1:** Examples of the condensation of meaning-bearing units into codes.

Meaning Units	Condensed Meaning Unit	Codes
After this, I learned how agile I was and what strength I had, which made me start to take more interest afterwards.	Got to learn how agile and strong I was; it made me interested afterwards	Learned about agility, strength, got interested
Depends on what your everyday life looks like. Do you sleep well? Do you have something to look forward to? Hobbies? If you don’t, there will be more ill health.	What does everyday life look like? Do you sleep well? Have something to look forward to? Hobbies?	Sense of participation and meaningfulness
Since you were not allowed to play handball, were not allowed to play matches, you felt no motivation, had nothing to train for, found no motivation.	You didn’t get to play games, you didn’t feel any motivation, nothing to train for	Not competing, no motivation, nothing to train for

**Table 2:** Sub-categories, categories, and themes abstracted from the meaning-bearing entities.

Theme	Sense of Participation and Autonomy Regarding Health					
Category	Satisfaction	Goal Formulation	Disappointment	Health Awareness	Limitations	Health-Promoting Change
Sub-category	Everyday well-being Increased motivation Lifestyle factors	Creation of meaningful lifestyle plan	Everyday ill-being	Comforting support from teachers, staff, and classmates Visualization of lifestyle factors FMS visualizes improvement areas	National restrictions due to pandemic COVID-19 restrictions make it more difficult to interact with classmates Activity limited in time without progression Difficult to talk to peers about lifestyle factors	Behavior changes regarding lifestyle factors

## Data Availability

The dataset supporting the conclusion of this article is available from the authors upon reasonable request and the completion of a data transfer agreement.
